# Selenium Supplementation does not Decrease Thyroid Peroxidase Antibody Concentration in Children and Adolescents with Autoimmune Thyroiditis

**DOI:** 10.1100/tsw.2010.91

**Published:** 2010-06-01

**Authors:** W. Bonfig, R. Gäertner, H. Schmidt

**Affiliations:** ^1^ Pediatric Endocrinology, Dr. Von Haunersches Children's Hospital, Germany; ^2^ Endocrinology, Internal Medicine, Medical Department — Innenstadt, Ludwig Maximilians University, Munich, Germany

**Keywords:** autoimmune thyroiditis, hypothyroidism, selenium supplementation, puberty

## Abstract

In adults, selenium supplementation decreases thyroid peroxidase antibody (TPO Ab) concentrations in patients with autoimmune thyroiditis (AIT). Our aim in this study was to investigate if selenium supplementation decreased TPO Ab and thyroglobulin antibody (Tg Ab) concentrations in children with AIT. Forty-nine patients (33 females) with newly diagnosed AIT and hypothyroidism were randomized to daily oral therapy with levothyroxine alone (group A, n = 18), levothyroxine plus 100 µg sodium-selenite (group B, n = 13), or levothyroxine plus 200 µg sodium-selenite (group C, n = 18). Mean age at diagnosis was 12.2 ± 2.2 years. All 49 patients needed a mean levothyroxine dose of 1.6 ± 0.5 µg/kg body weight to lower TSH to the treatment goal of 1–2 µU/ml, with no significant difference between groups. At study entry and after 12 months, TPO Ab concentrations were comparable in all three groups. Tg Ab concentrations decreased significantly after 12 months in group A and group C (*p* = 0.03 and *p* = 0.01), but not in group B (*p* = 0.06). It is our conclusion that selenium supplementation with sodium-selenite does not decrease TPO Ab concentrations in children and adolescents, neither given in the reduced dose of 100 µg daily nor given in the “adult” supplementation dose of 200 µg daily.

## INTRODUCTION

Autoimmune thyroiditis (AIT) is a common disease affecting more than 10% of adult females and 2% of adult males, but only about 3.5% of children. There is an atrophic and a hypertrophic course of the disease, and patients may be euthyroid, hypothyroid, or even transient hyperthyroid at onset of the disease. There is a genetic background, so patients with the human leukocyte antigens DR3 and DR5 are more likely to develop AIT. However, environmental factors, such as viral infections, iodine intake, and immunotherapeutic agents, also must be taken into account[1].

Selenium is an essential trace mineral for the proteogenomic synthesis of selenoproteins. Until now, 25 selenoproteins are known, most of which are enzymes. The thyroid is the organ with the highest selenium concentration due to the high content of selenoenzymes, which are important for thyroid hormone synthesis and metabolism. Severe iodine or selenium deficiency causes myoedematous cretinism because of damage to the thyroid[2]. The main enzymes within the thyroid are the deiodinases and glutathione peroxidases[3]. Selenium is also important for normal immune function and selenium deficiency is accompanied by loss of immune competence. Impairment in both cell-mediated immunity and B-cell function have been reported[4,5]. In addition, selenoenzymes act as an antioxidant and reduce free radical formation[6]. Possible therapeutic effects of selenium have already been shown in adults with rheumatoid arthritis[7].

Both mild iodine deficiency and selenium deficiencies have been reported in Germany[8,9]. Sources of selenium are crab, other shellfish, and fish. As selenium deficiency may influence both the immune response and the peroxidation of thyroid cell components, it has previously been studied if selenium supplementation has an influence on the natural course of Hashimoto thyroiditis in adults[10,11,12,13,14]. A significant decrease in thyroid peroxidase antibody (TPO Ab) concentration has been found in adults with a daily supplementation of 200 µg selenium. In an adult study on selenium treatment with variable doses (100 vs. 200 µg selenium) in AIT, Turker et al.[12] found that for suppression of serum concentrations of TPO Ab, selenomethionine doses higher than 100 µg daily were required.

The aim of this study was to investigate the effect of selenium supplementation on antibody concentrations and the dose-effect relationship in children and adolescents.

## PATIENTS AND METHODS

Patients were recruited in our pediatric endocrine outpatient clinic between the years 2006 and 2008. All children and adolescents with newly diagnosed AIT (positive thyroglobulin antibody [Tg Ab] and/or TPO Ab) and hypothyroidism with or without a goiter, and willing to take part in the study, were randomized to either levothyroxine treatment alone, levothyroxine plus 100 µg sodium-selenite, or levothyroxine plus 200 µg sodium-selenite. Patients were assigned to each treatment group in strict rotation starting with levothyroxine alone (group A), levothyroxine plus 200 µg sodium-selenite (group C), and levothyroxine plus 100 µg sodium-selenite (group B). No placebo was given in group A. Exclusion criteria were other autoimmune disease (i.e., type 1 diabetes mellitus), ongoing levothyroxine treatment or other constant medication, or other chronic disease. In all patients, typical hypoechogenicity of thyroid tissue was observed using high-resolution ultrasonography (12 MHz, Philips iU22, Hamburg, Germany). The treatment target for levothyroxine supplementation was a TSH concentration in the lower range between 1 and 2 µU/ml. All patients were otherwise healthy and did not receive any additional treatment, particularly no immunosuppressant or anti-inflammatory agents. Patients were followed quarterly by one experienced pediatric endocrinologist. Blood samples were drawn on each visit. For financial and logistical reasons, selenium concentration was unfortunately not measured before and during treatment. Patients and parents were highly motivated and compliant. There was no evidence of noncompliance with levothyroxine intake and, therefore, we conclude that patients were also compliant with sodium-selenite intake, as assured by parents who supervised regular intake. Patients also had to return empty blister packages of the study medication to double-check regular tablet intake. The patients were prospectively followed and data were collected on each visit.

Plasma total TPO Ab and Tg Ab were measured by a commercial enzyme luminescence assay (LIAISON, BRAHMS, Hennigsdorf, Germany; normal range TPO Ab and Tg Ab: <40 IU/ml), and free T3/free T4 (normal range free T3: 2.6–5.0 pg/ml, free T4: 0.8–1.8 ng/dl) and TSH concentrations (normal range: 0.3–4.0 µU/ml) were measured by an enzyme immunometric assay (ADVIA Centaur XP Immunoassay System, Siemens, Erlangen, Germany). The primary end point of the study was the change in TPO Ab concentration achieved with different doses of sodium-selenite plus levothyroxine supplementation. Response to treatment was defined as negative TPO Ab concentration after 1 year of levothyroxine ± sodium-selenite treatment. Secondary end points were Tg Ab concentration, prevalence of goiter in hypothyroid AIT, and levothyroxine doses needed to lower TSH concentration to the treatment goal between 1 and 2 µU/ml. The study protocol was approved by a local ethics committee and informed consent was obtained by both patients and their parents.

Statistical analysis was performed with the nonparametric Kruskal-Wallis test for between-group comparisons and with the nonparametric Wilcoxon test for longitudinal comparison of antibodies titers within each group. Statistical analysis was done with the SPSS 15.0 software (SPSS Inc., Chicago, 2008). A $p$ value < 0.05 was considered statistically significant.

## RESULTS

In this nonblinded, prospective study, 49 children and adolescents (16 males/33 females) with newly diagnosed AIT were included. Mean age at diagnosis was 12.2 ± 2.2 years (range: 7.6–16.4 years). In 61% (30 out of 49 patients), the thyroid gland was enlarged at the time of diagnosis. All patients were hypothyroid at diagnosis with a mean TSH concentration of 19.6 ± 42 µU/ml (median: 6 µU/ml) and therefore treated with levothyroxine. At the time of diagnosis, TPO Ab levels were positive with a mean concentration of 678 IU/ml in 43 out of 49 patients (88%). In six patients, only Tg Ab levels were positive (12%) at diagnosis (mean concentration: 864 IU/ml). Altogether, 18 patients were treated with levothyroxine alone (group A: 14 females/four males), 13 patients received levothyroxine plus 100 µg sodium-selenite (group B: nine females/four males), and 18 patients were treated with levothyroxine and 200 µg sodium-selenite (group C: 10 females/eight males), the supplementation dose used in adults. No side effects of levothyroxine and selenium treatment were observed during the study period.

Table 1 summarizes the results: age, weight, and TSH concentration at diagnosis were comparable between the three groups. All 49 patients needed a mean levothyroxine dose of 1.6 ± 0.5 µg/kg body weight to lower TSH concentration to the treatment goal of 1–2 µU/ml, with no significant difference between groups (range: 0.6–2.3 µg/kg body weight). With levothyroxine treatment, TSH decreased significantly in all groups (group A: $p = 0.001$, group B: $p = 0.005$, and group C: $p = 0.004$). At study entry and after 12 months, TPO Ab concentrations were comparable in all three groups. No statistically significant decrease in TPO Ab was observed after 12 months in all groups (group A: $p = 0.59$, group B: $p = 0.86$, group C: $p = 0.93$) (Fig. 1). Thus, sodium-selenite supplementation does not decrease TPO Ab concentration in children and adolescents, neither given in the reduced dose of 100 µg daily nor given in the supplementation dose of 200 µg used in adults with the same condition. However, the decrease in TPO Ab concentration was greatest in group C (reduction of 40.5%), but failed significance because of the small number and wide variation of TPO Ab concentration. Looking at the patient characteristics of "responders" and "nonresponders" to selenium treatment in group C, one can draw the conclusion that younger females are less likely to respond to selenium supplementation compared to older males ("nonresponders": eight females, two males, mean age at diagnosis 10.6 years [median: 10.5 years] compared to "responders": two females, six males, mean age at diagnosis 12.5 years [median: 12 years], $p < 0.05$ for age at diagnosis).

Unexpectedly, Tg Ab concentration decreased significantly after 12 months in group A and group C ($p = 0.03$ and $p = 0.01$), but not in group B ($p = 0.06$) (Fig. 2). In group B, significance failed because the number of subjects was lower and because there were two outliers in that group that seemed to affect the statistical results.

A secondary end point of our study was to determine the necessary levothyroxine dose in hypothyroid AIT in children and adolescents to lower TSH below 2 µU/ml. The mean daily levothyroxine dose was 1.6 ± 0.5 µg/kg body weight to achieve this treatment goal.

**TABLE 1**
**Results of the Three Different Treatment Groups (Mean ± SD)**

| | Group A (n = 18) | Group B (n = 13) | Group C (n = 18) | *p* Value |
|---|---|---|---|---|
| Sodium-selenite supplementation [µg/day] | 0 | 100 | 200 | 0.00 |
| Age at diagnosis [years] | 12.5 ± 1.9 | 12.7 ± 2.2 | 11.5 ± 2.3 | 0.19 |
| Weight [kg] | 43.7 ± 10.7 | 52.4 ± 13.1 | 41.6 ± 12.6 | 0.07 |
| Levothyroxine dose/body weight [µg/kg] | 1.7 ± 0.6 | 1.6 ± 0.5 | 1.7 ± 0.4 | 0.62 |
| TSH at diagnosis [µU/ml] | 13.1 ± 16.4 | 35.4 ± 74 | 15.4 ± 27.7 | 0.51 |
| TPO Ab at diagnosis [U/ml] | 656 ± 698 | 658 ± 868 | 715 ± 1012 | 0.99 |
| Tg Ab at diagnosis [U/ml] | 518 ± 676 | 815 ± 1250 | 1245 ± 1914 | 0.49 |
| TSH at 12 months [µU/ml] | 1.9 ± 1.7 | 1.8 ± 1.4 | 2.1 ± 2 | 0.99 |
| TPO Ab at 12 months [U/ml] | 518 ± 526 | 510 ± 499 | 426 ± 520 | 0.83 |
| Tg Ab at 12 months [U/ml] | 329 ± 404 | 669 ± 1363 | 671 ± 1347 | 0.38 |

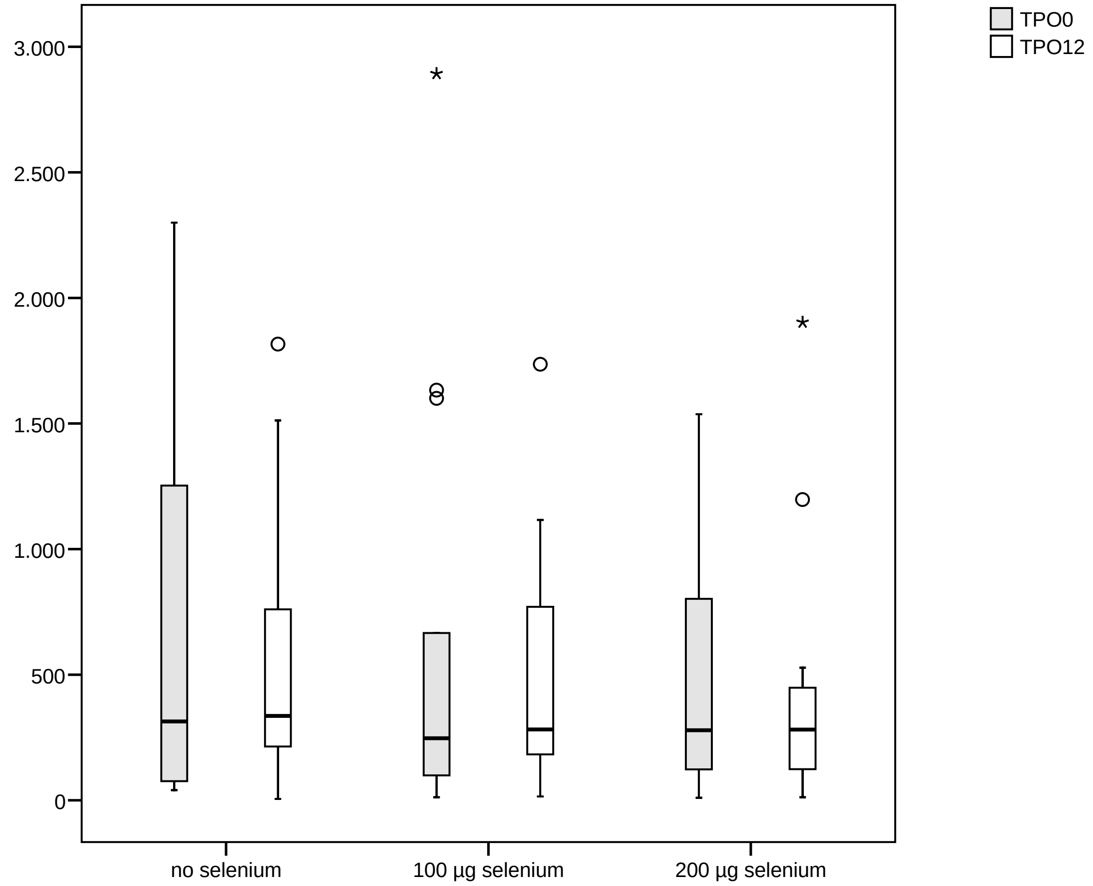

**FIGURE 1.** Boxplot (median, first, and third quartile, whiskers 3rd/97th percentile) showing TPO Ab titers [U/ml] at diagnosis and after 12 months. The change in TPO Ab titers is statistically not significant.

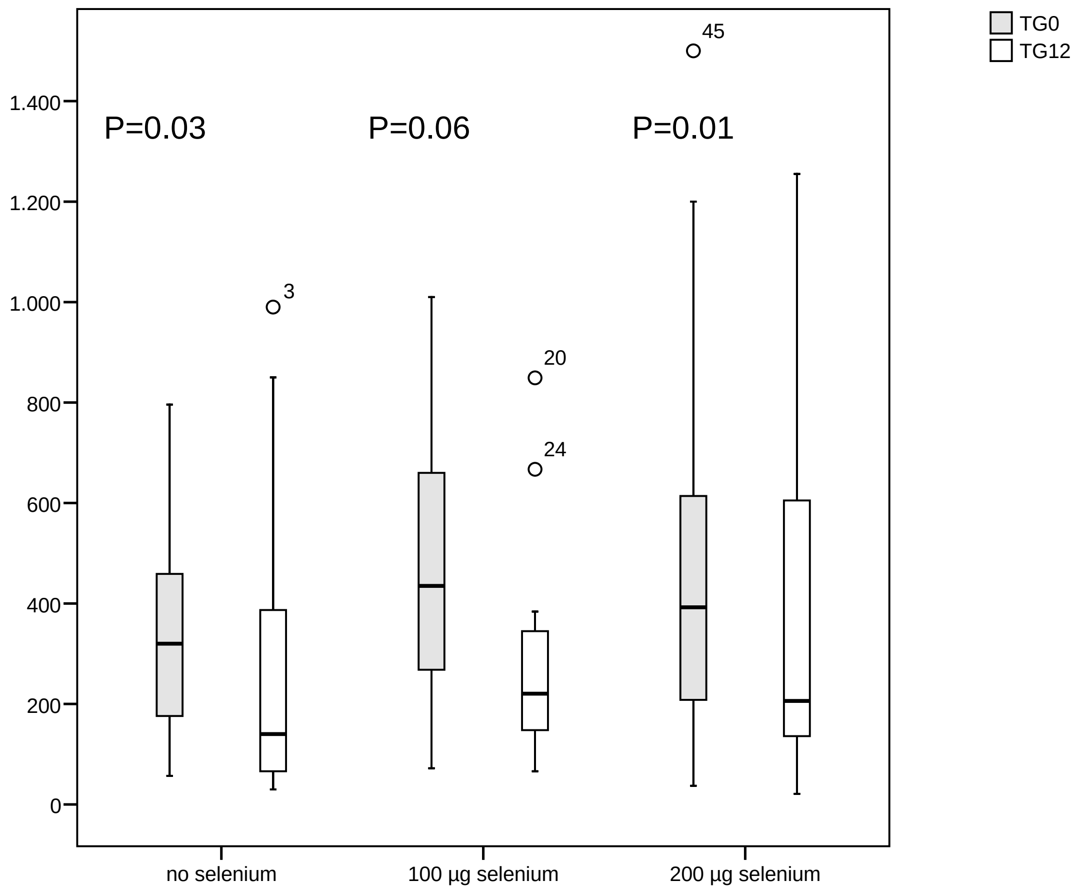

**FIGURE 2.** Boxplot (median, first, and third quartile, whiskers 3rd/97th percentile) showing Tg Ab titers [U/ml] at diagnosis and after 12 months. Significant decrease in Tg Ab concentration (no selenium [group A] and 200 µg selenium [group C]).

## DISCUSSION

This is the first randomized prospective study to investigate if variable doses of sodium-selenite supplementation influence the course of AIT in children and adolescents. We did not observe a significant decrease in thyroid-specific TPO Ab concentrations in children and adolescents with AIT, neither with 100 µg nor with 200 µg selenium supplementation daily. This result is contradictory to the effect observed in several studies in adults[10,11,12,13]. One could speculate that the autoimmune inflammation is extremely active during puberty, so that selenium supplementation does not have enough potency to overcome this highly active autoimmune process. This thesis is supported by the fact that only one patient in this study was able to stop treatment after 1 year without relapse.

In an adult study on selenium treatment with variable doses (100 vs. 200 µg selenium) in AIT, Turker et al.[12] found that for suppression of serum concentrations of TPO Ab, selenomethionine doses higher than 100 µg daily were required.

Selenium-dependent enzymes have diverse effects both within the thyroid gland and also on the immune system. It has been shown that with severe selenium deficiency, glutathione peroxidase activity decreases and thereby may contribute to oxidative damage of the thyroid cell[15]. In a rat model, selenium supplementation prevents this oxidative damage. Therefore, early treatment in the course of AIT may save more thyrocytes. If started late, selenium supplementation may be ineffective in the atrophic course of AIT. In our cohort of children and adolescents, we found a relatively high prevalence of goiter in AIT (61%) and therefore would have expected a conspicuous effect of selenium treatment.

In adults, a beneficial effect of selenium supplementation of 200 µg daily was observed in double blind studies in patients with rheumatoid arthritis[7] and, in higher doses (500–1000 µg), even in patients with severe sepsis[16]. These effects are explained by the cellular immune response driven by selenium: increased production of interferon gamma and other cytokines, early peak T-cell proliferation, and an increase in T-helper cells[17].

The decrease of Tg Ab concentration in our study seems to be unrelated to the therapeutic effect of selenium, since there is no pathophysiologic explanation for this interaction and since such an effect has not been observed in previous studies. It is more likely that the decrease in Tg Ab is an effect of levothyroxine treatment. In general, Tg Ab are less specific for AIT, because Tg is a physiologically circulating antigen and therefore is not necessarily an antigen only expressed during thyroid-specific autoimmune inflammation. Therefore, Tg Ab concentrations are usually less specific for the diagnosis and course of AIT, but after all, 12% of patients in our cohort of children and adolescents had only positive Tg Ab and negative TPO Ab, together with typical ultrasonographic findings and hypothyroidism at the time of diagnosis. Thus, in these six patients, no decrease in TPO Ab concentration was possible during follow-up, while Tg Ab concentration decreased with levothyroxine (± sodium-selenite) treatment.

A secondary end point of our study was to determine the necessary levothyroxine dose in hypothyroid AIT in children and adolescents to lower TSH below 2 µU/ml. To our knowledge, such data have not been published so far. The mean levothyroxine dose of 1.6 µg/kg body weight in AIT is lower than the general recommendation for levothyroxine substitution in congenital hypothyroidism during puberty (2–3 µg/kg body weight), but is equal to the recommended replacement dose in adults with congenital hypothyroidism (1.6–1.8 µg/kg “ideal” body weight)[18].

In conclusion, no significant impact of high- or low-dose sodium-selenite supplementation on inflammatory activity in AIT in children and adolescents has been observed in a 1-year period. Further studies with determination of pre- and post treatment selenium concentrations, with larger patient numbers, and the higher selenium dosage (200 µg daily) in children and adolescents are needed to verify these preliminary results.
